# The ribonuclease polynucleotide phosphorylase can interact with small regulatory RNAs in both protective and degradative modes

**DOI:** 10.1261/rna.052886.115

**Published:** 2016-03

**Authors:** Katarzyna J. Bandyra, Dhriti Sinha, Johanna Syrjanen, Ben F. Luisi, Nicholas R. De Lay

**Affiliations:** 1 Department of Biochemistry, University of Cambridge, Cambridge CB2 1GA, United Kingdom; 2 Department of Microbiology and Molecular Genetics, University of Texas Medical School, Houston, Texas 77030, USA; 3 Graduate School of Biomedical Sciences, University of Texas Health Science Center, Houston, Texas 77030, USA

**Keywords:** small regulatory RNA, Hfq, RNA degradation, polynucleotide phosphorylase, RNase E

## Abstract

In all bacterial species examined thus far, small regulatory RNAs (sRNAs) contribute to intricate patterns of dynamic genetic regulation. Many of the actions of these nucleic acids are mediated by well-characterized chaperones such as the Hfq protein, but genetic screens have also recently identified the 3′-to-5′ exoribonuclease polynucleotide phosphorylase (PNPase) as an unexpected stabilizer and facilitator of sRNAs in vivo. To understand how a ribonuclease might mediate these effects, we tested the interactions of PNPase with sRNAs and found that the enzyme can readily degrade these nucleic acids in vitro but, nonetheless, copurifies from cell extracts with the same sRNAs without discernible degradation or modification to their 3′ ends, suggesting that the associated RNA is protected against the destructive activity of the ribonuclease. In vitro, PNPase, Hfq, and sRNA can form a ternary complex in which the ribonuclease plays a nondestructive, structural role. Such ternary complexes might be formed transiently in vivo, but could help to stabilize particular sRNAs and remodel their population on Hfq. Taken together, our results indicate that PNPase can be programmed to act on RNA in either destructive or stabilizing modes in vivo and may form complex, protective ribonucleoprotein assemblies that shape the landscape of sRNAs available for action.

## INTRODUCTION

Small noncoding regulatory RNAs (sRNAs) are versatile regulators of gene expression throughout all branches of life (Wagner and Romby 2015). These molecules can change gene expression by altering mRNA stability and/or translational efficiency, and they achieve specificity for defined transcripts through complementary base-pairing (Thomason and Storz 2010; Pasquinelli 2012; De Lay et al. 2013). In the bacteria *Escherichia coli* and *Salmonella enterica*, the exoribonuclease polynucleotide phosphorylase (PNPase) has been shown to degrade certain sRNAs, resulting in changes in gene expression (Viegas et al. 2007; Andrade and Arraiano 2008; Andrade et al. 2012). An analogous activity of the human PNPase has been implicated in the control of microRNAs, a class of eukaryotic sRNAs (Das et al. 2010).

PNPase is a 3′ to 5′ phosphorolytic exoribonuclease and its activity has been elucidated through extensive biochemical, structural, and genetic studies (for review, see Andrade et al. 2009). The catalytic core bears two ancient RNase PH-like domains, separated by an alpha helical domain, which present interfacial surfaces supporting trimerization to form a toroidal architecture with a central channel within which the active sites reside (Symmons et al. 2000). Two nucleic acid binding domains at the C terminus, belonging to the conserved KH and S1 families, decorate the entrance to a central pore. Once engaged by those domains, the RNA threads into the central pore to one of the active sites; there, its 3′ terminal phosphodiester bond can be cleaved by a phosphate molecule orientated for attack. PNPase acts processively to degrade the RNA in the 3′ to 5′ direction, releasing nucleoside diphosphate products as it proceeds. Previous in vitro studies of the *E. coli* PNPase have shown that the S1 and KH domains and the core play important roles in substrate capture (Stickney et al. 2005; Matus-Ortega et al. 2007; Wong et al. 2013), and recent evidence supports a model for S1 contributing to PNPase auto-regulation by binding the 5′ UTR and repressing translation (Robert-Le Meur and Portier 1994; Carzaniga et al. 2015).

Recent reports have identified an unexpected effect of PNPase in *E. coli* in stabilizing certain sRNAs, which appears antithetical to a role as a purely degradative exoribonuclease. Deletion of the gene encoding PNPase (*pnp*) resulted in destabilization of several sRNAs including CyaR and RyhB, which are involved in response to nutrient sensing and regulation of iron-storage proteins, respectively (De Lay and Gottesman 2011). Using a translational reporter fusion to an mRNA target of CyaR, the *ompX* transcript encoding an outer membrane porin, it was also demonstrated that deletion of *pnp* resulted in increased OmpX expression (De Lay and Gottesman 2011). Altogether these results suggested that the reduced sRNA levels in the absence of *pnp* contributed to a reduction in sRNA-mediated regulation of gene expression.

The defect in sRNA stability in a Δ*pnp* strain was also observed in a subsequent study that found that PNPase-mediated stabilization of some sRNAs was growth phase dependent (Andrade et al. 2012). Interestingly, global analyses have shown that the levels of many transcripts are reduced when PNPase expression is decreased in human melanoma cells (Sokhi et al. 2013) and *E. coli* (Mohanty and Kushner 2003), indicating that the stabilizing effect of the enzyme might be more general and not restricted to regulatory RNAs. It was unclear from the available data whether the genetic effects of PNPase on sRNAs was through a direct protection or an indirect mechanism, e.g., by reducing the levels of other ribonucleases or cofactors including repressive RNAs that might normally contribute to the degradation of these sRNAs. It is interesting in this regard that depletion of another exoribonuclease in *E. coli*, RNase II, stabilizes a subset of transcripts by trimming of the 3′ end, which in turn appears to block the binding of another RNase that would degrade it (Mohanty and Kushner 2003).

Here, we have further explored the mechanism by which *E. coli* PNPase protects certain sRNAs from degradation. We demonstrate that *E. coli* PNPase can sequester some sRNAs without degrading them, and that PNPase impacts the distribution of sRNAs associated with Hfq in vivo. We provide evidence for a ribonucleoprotein complex between Hfq, PNPase, and sRNA and show that within this complex the sRNA is protected against the action of PNPase itself and possibly other enzymes. Additional proteins or RNAs or regulatory ligands (Nurmohamed et al. 2011) may in principle participate in this complex in vivo. The sequestration function of PNPase is a novel activity for this highly conserved, well-studied exoribonuclease.

## RESULTS

### PNPase associates with sRNAs that it protects from degradation

Deletion of *pnp* from *E. coli* caused a decrease in the stability of several sRNAs including SgrS, RyhB, and CyaR (De Lay and Gottesman 2011). We explored if sRNAs dependent on PNPase for stability are physically associated with this protein in vivo, using an affinity-tagged enzyme. For these experiments, the chromosomal copy of *pnp* was replaced with a C-terminal 3xFLAG tagged allele of PNPase by lambda Red-mediated recombineering, generating a strain that has the sequence encoding the epitope tag at the 3′ end of *pnp*, but has no additional alterations at this chromosomal locus.

The addition of the 3xFLAG-tag to PNPase does not appear to alter its cellular activities. One measure for PNPase activity in vivo is the extent of LeuX transcript processing (Mohanty and Kushner 2010), and, consistent with previous findings, we found that the primary transcript accumulates in a *pnp* deletion mutant of *E. coli* (Supplemental Fig. S1A). Using Northern blot analysis, we observed efficient processing of the primary LeuX transcript in the strain expressing PNPase-FLAG compared to the wild type (78% of wild type), indicating that the FLAG-tagged version of PNPase is functional for this process (Supplemental Fig. S1A, upper panel). Another assay for PNPase activity is its contribution to the stability and activity of CyaR (De Lay and Gottesman 2011). We observed a 40% decrease in the level of CyaR in the *pnp* deletion mutant from exponential phase cultures as compared to the wild-type *E. coli* strain, whereas the PNPase-FLAG encoding strain had nearly wild-type CyaR levels (98% of wild type; Supplemental Fig. S1A, lower panel), consistent with it contributing to sRNA stabilization. Deletion of *pnp* resulted in a 35% decrease in the ability of CyaR to regulate its target mRNA, the *ompX* reporter fusion, but the sRNA was able to negatively regulate its target mRNA *ompX* in the PNPase-FLAG encoding strain (Supplemental Fig. S1B), indicating that sRNA-mediated regulation of gene expression was also maintained. Notably, a Δ*hfq* strain shows a nearly identical defect in sRNA-mediated regulation as a Δ*pnp* strain under the conditions used in this assay, indicating that the two proteins make comparable contributions to the regulation (Supplemental Fig. S1C).

**FIGURE 1.**
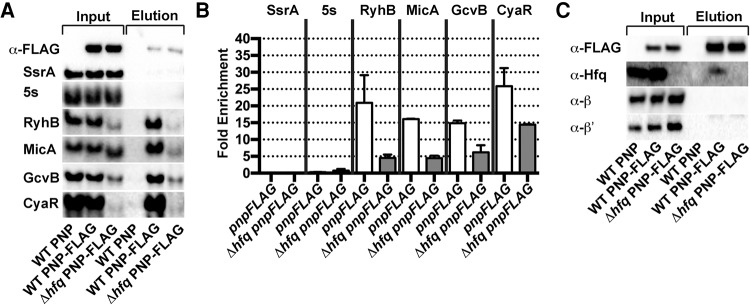
PNPase physically associates with sRNAs and Hfq. (*A*) Northern blot analysis of RNAs that coimmunoprecipitated with affinity-tagged PNPase. PNPase-FLAG was immunoprecipitated from cell extracts prepared from late exponential phase cultures of a wild type (KR10000; WT) and derived strain expressing PNPase-FLAG (NRD1243; *pnpFLAG*) or the derived *hfq* deletion mutant (NRD1297; Δ*hfq pnpFLAG*) using an anti-FLAG M2 agarose resin. For Western blots probed with anti-FLAG antibody, the ratio of protein samples from input and elution loaded was 1:1, and for Northern blots, the ratio of the volume of input to elution loaded was 1:162. SsrA and 5S rRNA are known not to be Hfq/PNPase dependent. (*B*) Fold enrichment for each RNA upon immunoprecipitation of PNPase. Fold enrichment of a given RNA upon immunoprecipitation was determined by first calculating the signal intensity per nanogram of RNA for the input and the elution from the Northern blots in *A*. The normalized elution signal was then divided by the input signal. (*C*) Western blot analysis of proteins that copurified with affinity-tagged PNPase. PNPase-FLAG was immunoprecipitated as described in *A*, except formaldehyde crosslinking was performed prior to purification and crosslinks were reversed post purification. Western blots were probed with antibodies against the 3xFLAG peptide, Hfq, β (RpoB), and β′ (RpoC).

Having shown that the affinity tag does not significantly affect PNPase action in vivo, we assessed whether PNPase-FLAG and sRNAs are physically associated using coimmunoprecipitation assays with lysates from late exponential phase cultures (OD_600_ ∼ 1.0) of a wild-type *E. coli* strain or strain encoding PNPase-FLAG. sRNAs including GcvB, RyhB, CyaR, and MicA were readily detected by Northern blot in both the input and elution fractions from cell lysates prepared from the PNPase-FLAG expressing strain, and were enriched upon immunoprecipitation by PNPase by 15-, 20-, 27-, and 16-fold, respectively (Fig. 1). In control experiments with a strain expressing the untagged PNPase, no sRNAs were detected in the elution fraction. The coprecipitation of the sRNAs with PNPase does not appear to be due to indiscriminate association with bulk RNA, because there is not significant association of other species that are abundant in the cell extracts such as 5s rRNA and SsrA (tmRNA), a noncoding RNA involved in rescuing stalled ribosomes (Muto et al. 1996).

Surprisingly, the sRNAs associated with PNPase appeared to be full-length, based on migration in denaturing gel electrophoresis. It remained possible that the RNAs detected in the input and elution fractions differed by a few nucleotides, which would not be resolved by the 10% TBE-urea polyacrylamide gel; however, no discernible degradation (or lengthening) of the sRNAs coimmunoprecipitating with PNPase was observed.

Given these results, we examined more globally the RNA species that coimmunoprecipitated with PNPase. We performed high-throughput sequencing of RNA isolated from cells from the same culture before and after coimmunoprecipitation with PNPase-FLAG. sRNAs are the preferred species stably associated with PNPase. Of the 24 known Hfq-binding sRNAs that were detected, 11 (46%) were enriched by more than twofold in the PNPase co-IP, that is, more than twice the number of paired end reads were detected in the immunoprecipitated RNA sample compared with the total RNA input of the pull down (Table 1). In contrast, only 6.3% of all mRNAs detected were enriched in the PNPase Co-IP (278 out of 4382; Supplemental Table S3).

**TABLE 1.**
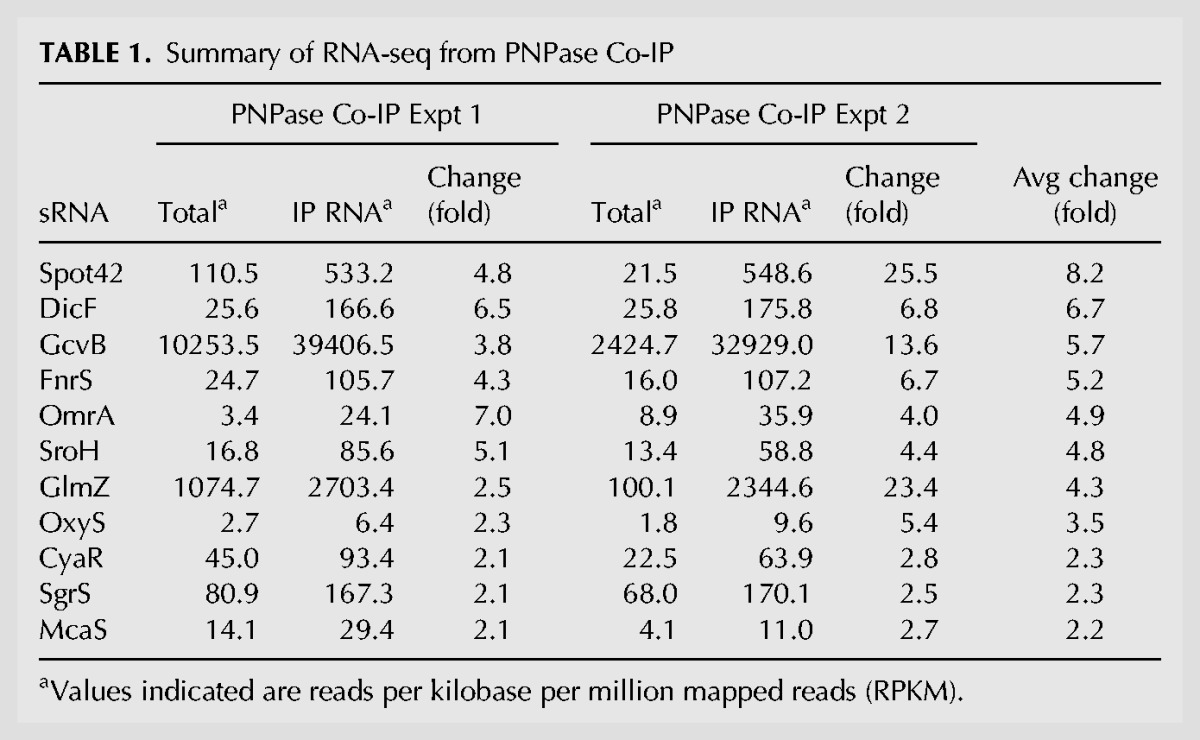
Summary of RNA-seq from PNPase Co-IP

We explored whether an inhibitor of PNPase activity might affect the population of associated RNAs. Tungstate was examined as a potential inhibitor of PNPase exoribonuclease activity, because the crystal structure of the enzyme revealed that the metal is bound in the catalytic center at the predicted phosphate binding site (Symmons et al. 2000). Indeed, at a concentration range of 2–10 mM sodium tungstate efficiently inhibited the exoribonuclease activity of PNPase in vitro even in the presence of phosphate (Fig. 5B, below) and did not significantly influence PNPase-sRNA binding (Fig. 3D, below). In most cases, tungstate did not substantially affect the total amount of sRNA immunoprecipitated with PNPase (Tables 1, 2), as determined by the normalized read count (RPKM). These results suggest that the sRNAs coimmunoprecipitated in the absence of tungstate are not simply selectively enriched as a consequence of a degradative process.

**TABLE 2.**
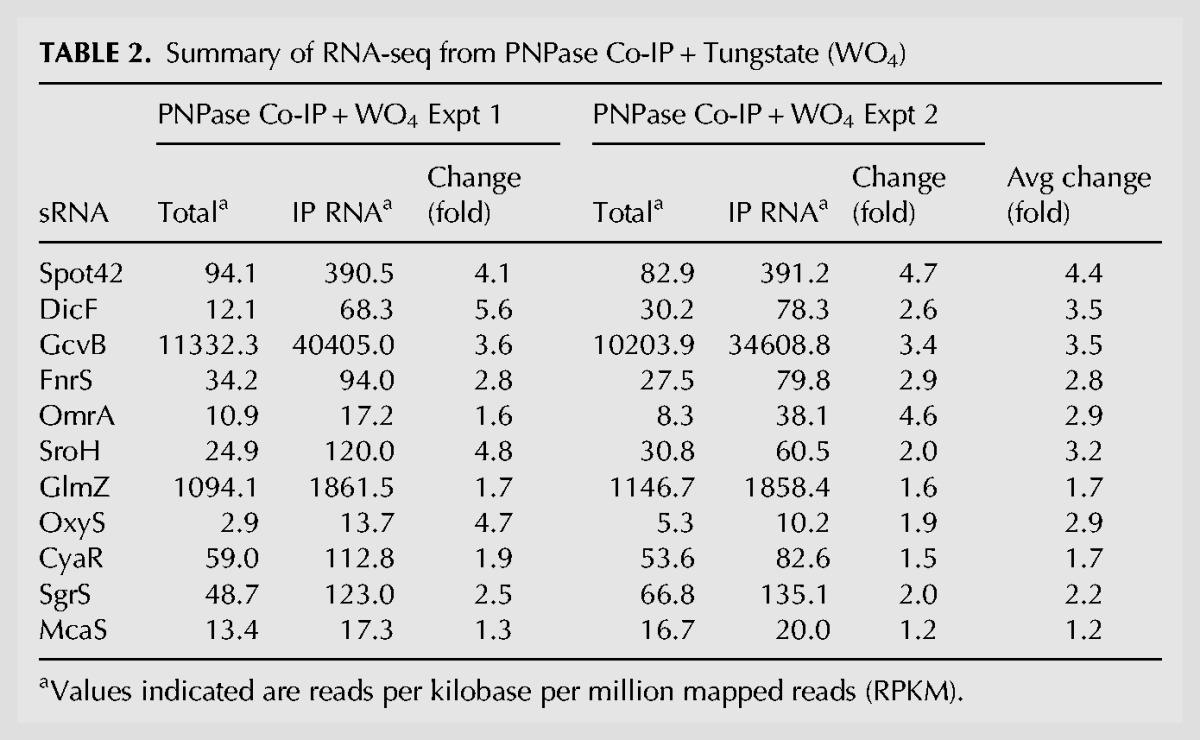
Summary of RNA-seq from PNPase Co-IP + Tungstate (WO_4_)

The fact that apparently full-length sRNAs coimmunoprecipitated with PNPase suggested to us that either PNPase was able to bind sRNAs, but not effectively degrade them, or that PNPase was forming a complex with sRNA bound to another protein or other RNA, which was providing protection from the exoribonuclease activity of PNPase. Several studies have shown that Hfq binds many sRNAs in *E. coli* and *S. enterica* (Zhang et al. 2003; Sittka et al. 2008; Bilusic et al. 2014), suggesting the possibility that PNPase binds sRNAs indirectly via Hfq. Thus, we first tested whether or not Hfq interacts with PNPase-FLAG via a coimmunoprecipitation assay. Consistent with previous findings that Hfq copurifies with PNPase (Mohanty et al. 2004; Butland et al. 2005; Obregon et al. 2015), Hfq coimmunoprecipitated with PNPase-FLAG (Fig. 1C; Supplemental Table S4). Next, we determined whether sRNAs coimmunoprecipitate with PNPase-FLAG in an *hfq* deletion strain. GcvB, CyaR, RyhB, and MicA still coimmunoprecipitated with PNPase-FLAG in the absence of Hfq, although there is less enrichment as compared to the *hfq*^*+*^ strain expressing PNPase-FLAG (Fig. 1B). RyhB and MicA recovered in the PNPase Co-IP were noticeably shorter in the *hfq* deletion mutant, which could be the result of substantial trimming of the 3′ end by PNPase in the absence of the chaperone. Regardless, these results are consistent with PNPase being able to associate with certain sRNAs without completely degrading them, and with the capacity of this enzyme to form complexes with Hfq that may be RNA mediated. The latter was explored by in vitro binding experiments described below.

### PNPase S1 domain and active site contribute to binding of RyhB in vivo

The results described above are consistent with a model in which PNPase directly binds sRNAs that it protects from degradation; however, it is unclear how exactly PNPase is interacting with these sRNAs. Previous studies have shown that the S1 domain of PNPase is critical for binding RNA substrates (Stickney et al. 2005; Fernandez-Ramirez et al. 2010; Hardwick et al. 2012), and it has been postulated that residues along the length of the central pore, including those within the active site, are also involved in RNA binding (Nurmohamed et al. 2009). We directly assessed the contribution of the S1 domain and the active site residues in sRNA binding both in vivo and in vitro.

**FIGURE 2.**
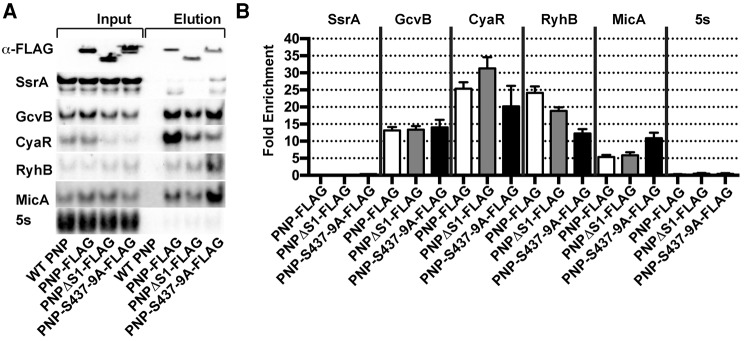
Association of PNPase with RyhB in vivo is affected by active site residues S437, S438, and S439 and the S1 domain. (*A*) Flag-tagged PNPase, or derived variants lacking the S1 domain or having three active site residues mutated (S437A S438A S439A) were used to coprecipitate sRNAs, which were analyzed by Northern blot. For Western blots, an equal volume of protein samples from input and elution were loaded, and for Northern blots, the ratio of input to elution loaded was 1:162. (*B*) Fold enrichment of RNA upon immunoprecipitation was calculated as in Figure 1.

We replaced the chromosomal copy of *pnp* in *E. coli* with an allele encoding a FLAG-tagged PNPase that either lacks the S1 domain or has alanine substitutions in the three active site residues S437, S438, and S439 postulated to bind the attacking phosphate that cleaves the RNA substrate (Nurmohamed et al. 2009). All of the resulting constructs expressed well and were soluble (Fig. 2A, top panel). Deletion of the S1 domain or mutation of the active site resulted in a substantial decrease in the steady-state level of CyaR, much like the behavior seen with the Δ*pnp* strain. Quantification of the samples revealed that CyaR levels in cell extracts of the S1 domain and active site mutants were reduced by >65% and 69%, respectively.

Deletion of the S1 domain or mutation of the active site of PNPase significantly decreased the amount of RyhB immunoprecipitated. However, other sRNAs responded differently to the PNPase mutations. Mutating the active site did not significantly change the amount of CyaR coimmunoprecipitating with PNPase (Fig. 2A,B), but increased MicA binding. Deletion of the S1 domain had no significant effect on MicA binding. Finally, deletion of the S1 domain or mutation of the active site did not appear to have a significant effect on the levels of GcvB coprecipitating with PNPase. These results indicate that the S1 domain and active site affect binding of RyhB, but it should be noted that they are not the sole determinants for binding that sRNA in vivo. The results also indicate that there may not be a common mode of interaction between PNPase and sRNAs, with each case being different in detail.

### Purified PNPase binds sRNAs with high affinity

**FIGURE 3.**
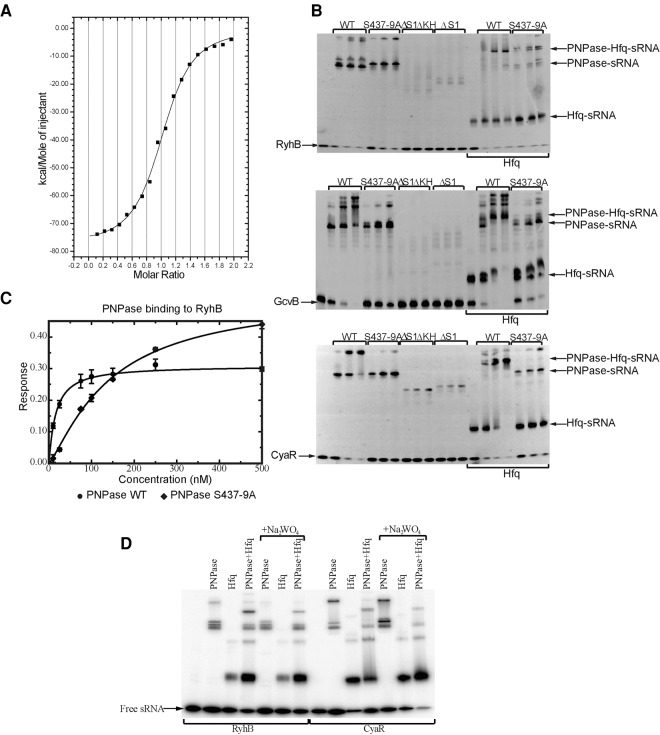
PNPase binds sRNAs directly and as a ternary complex with Hfq. (*A*) RyhB interactions with PNPase evaluated by isothermal titration calorimetry. All binding experiments were performed in the absence of phosphate to prevent phosphorylysis. The fitted binding parameters for the isotherm are *N* = 1.01 ± 8.5 × 10^−3^, *K* = 1.17 × 10^−7^± 9.4 × 10^−5^ M^−1^, ΔH = −7.17 × 10^−4^ ± 890.1 cal/mol, ΔS = −224 cal/mol/deg. (*B*) The PNPase/sRNA interactions require the S1 and KH domains and active sites. Electrophoretic mobility shift binding assays of PNPase (0.2, 0.4, and 0.6 μM) with RyhB, CyaR, and GcvB (0.2 μM) are shown. Binding experiments include deletion mutants of the KH and S1 RNA-binding domains of PNPase and the presence of Hfq (0.2 μM). (*C*) Binding of PNPase and active site mutant by surface binding analysis. The RNA with a 5′-biotinyl modification was immobilized on the streptavidin-coated detector tip of an Octet device and isotherms measured in solutions of varying protein concentration. (*D*) Gel shift mobility assays in the presence of 2 mM sodium phosphate and 10 mM sodium tungstate of RyhB and CyaR with PNPase (30 nM), Hfq (15 nM), and those two proteins combined.

While our coimmunoprecipitation experiments demonstrated that PNPase binds sRNAs in the presence or absence of Hfq, it remained possible that PNPase only interacts with sRNAs indirectly through another protein or RNA. To assess the ability of PNPase to directly bind sRNAs, we purified overexpressed recombinant PNPase and evaluated binding of several sRNAs in vitro. Firstly, we examined interaction of PNPase with RyhB, one of the sRNAs for which stability was shown to be PNPase dependent in vivo. Under non-catalytic conditions (namely, the absence of phosphate), interaction of RyhB and PNPase could be detected by isothermal titration calorimetry (ITC). Fitting the binding isotherm, RyhB and PNPase associate with 85 nM K_d_ and 1:1 stoichiometry of sRNA:PNPase trimer (Fig. 3A). The exothermic enthalpy change of −77.68 kcal/mol is large compared with other RNA–protein interactions measured by this method (Recht and Williamson 2004; McKenna et al. 2006), so it is possible that the binding of PNPase to RyhB also involves a coupled equilibrium with refolding of the RNA. As this enthalpy change is comparable to the values observed for sRNA binding to Hfq as examined by ITC (Mikulecky et al. 2004) and Hfq has known RNA remodeling activity (Zhang et al. 2002), the results suggest that RyhB undergoes a conformation change upon interacting with PNPase.

The coimmunoprecipitation experiments suggested that the S1 domain and active site of PNPase are both important for binding certain sRNAs such as RyhB. To more precisely examine the effects of deleting the S1 domain or mutating the active site on sRNA binding by PNPase, we purified the wild-type PNPase, the active site mutant (PNPase S347-9A), and the truncated form lacking the KH and/or S1 domain, and tested their binding to RyhB by electrophoretic mobility shift assays. Deletion of the S1 domain alone or in combination with the KH domain strongly reduced binding to this sRNA (Fig. 3B). Mutation of the active site serines also reduced the affinity for RyhB, suggesting a role of the active site in sRNA–PNPase interaction.

To evaluate quantitatively the difference in binding between wild-type and inactive mutant of PNPase, we measured binding isotherms using interferometry. For this experiment, the 5′ end of RyhB was labeled with biotin and immobilized on a streptavidin sensor tip. The binding of PNPase wild type and S347-9A mutant were assayed, resulting in estimated *K*_d_ of 15.8 ± 1.9 and 149.0 ± 13 nM, respectively (Fig. 3C), corroborating the diminished affinity of the mutant indicated by EMSA. The reduced affinity of the mutant PNPase for the sRNA is rather striking given the distance between the internal active site and the surface-presented KH and S1 domains. There is no evidence that the active site mutations would influence in any way the structure of the KH and S1 domains, but other studies have also shown that the active site channel contributes to the affinity for RNA substrates (Stickney et al. 2005).

EMSA assays also revealed that deleting the S1 domain or mutating the active site of PNPase decrease association with CyaR and GcvB (Fig. 3B). This in vitro behavior differs from the in vivo results from the co-IP shown in Figure 2. For CyaR, similar enrichments were found for the wild-type and mutant PNPases, but it is difficult to assess if these reflect similar extent of binding, as there were substantially reduced levels of CyaR in strains expressing PNPase-FLAG lacking the S1 domain or active site serines (Fig. 2). This could indicate that the majority of CyaR molecules did not bind the mutant PNPase-FLAG and were rapidly degraded. The in vitro experiments, which provide a more precise measure of binding than co-IPs, demonstrate that the S1 domain and active site contribute to sRNA interaction, but may not be the sole determinants in vivo.

### The PNPase–sRNA association contributes to PNPase-mediated protection

**FIGURE 4.**
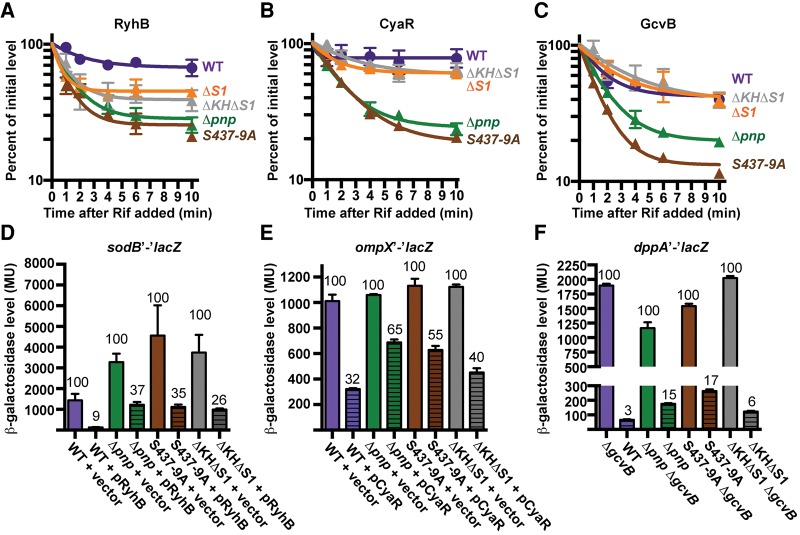
The active site and S1 domain of PNPase are both important for sRNA stabilization and target regulation. (*A*) Analysis of RyhB. A strain harboring a P_BAD_::*sodB′*-′*lacZ* fusion (WT, NRD537) or derived strains: Δ*pnp* (NRD1114), PNPase ΔKHΔS1 (NRD1116), PNPase ΔS1 (NRD1151), or PNPase S437-9A (NRD1117) were grown to exponential phase. RyhB expression was then induced from the chromosome by the addition of dipyridyl. Rifampicin was added after 15 min. (*B*) Analysis of CyaR. Transcription of the sRNA was induced from a *lac-*based promoter on a plasmid using a Δ*cyaR* strain carrying P_BAD_::*ompX′-′lacZ* (WT, NRD377), or derived mutants (Δ*pnp*, NRD677; ΔKH ΔS1, NRD1044; ΔS1, NRD1147; S437-9A, NRD1045). (*C*) Analysis of GcvB. The sRNA was expressed from its native chromosomal locus in the strains from *A*. Northern blots were performed in duplicate and quantified with standard error of mean (SEM) indicated for RyhB (*A*), CyaR (*B*), or GcvB (*C*). Strains from *A* and *B* harboring an empty vector or a plasmid expressing the negatively regulating sRNAs, RyhB (*D*), or CyaR (*E*), respectively, were used in a β-galactosidase assay. (*F*) β-Galactosidase assays were performed on a wild-type or Δ*gcvB* strain harboring a P_BAD_::*dppA′-′lacZ* fusion or derived strains (WT, NRD1382; Δ*pnp*, NRD1383; ΔKH ΔS1, NRD1384; S437-9A, NRD1385; Δ*gcvB*, NRD1388; Δ*pnp* Δ*gcvB*, NRD1389; ΔKH ΔS1 Δ*gcvB*, NRD1390; S437-9A Δ*gcvB*, NRD1391). The percentage of β-galactosidase activity relative to the strain lacking expression of the given sRNA is indicated *above* each bar.

Next we examined whether portions of PNPase important for sRNA binding in vitro were required for sRNA stabilization and function in sRNA-mediated regulation of gene expression. We compared the stability of RyhB, CyaR, and GcvB in strains that are *pnp* null or express wild type, ΔS1, or S437-9A PNPase. We also examined the behavior of strains with PNPase lacking both KH and S1 domains (PNPaseΔKHΔS1). In these experiments, sRNAs were induced for 15 min in exponential phase to deplete the pool of target mRNAs (Masse et al. 2003), and then rifampicin was added to block transcription of both the sRNA and its partner mRNAs. With this experimental design, target pairing should not significantly contribute to sRNA decay. The decay of each sRNA (RyhB, CyaR, and GcvB) was initially rapid in the *pnp* deletion strain, but then plateaued leaving >20% of the original sRNA pool remaining. This biphasic decay in the *pnp* deletion strain indicates that there are at least two reservoirs of sRNAs present in *E. coli*, a large population that is dependent upon PNPase for stability and a smaller pool that is not. The stabilities of RyhB, CyaR, and GcvB were also found to be dramatically reduced when the PNPase active site was mutated as compared to the wild-type strain. For RyhB and CyaR, these stabilities were comparable to that observed in the *pnp* deletion strain, but for GcvB the destabilization was even greater for the active site mutant (Fig. 4A–C). Deletion of the S1 domain of PNPase had differing effects on these three sRNAs, resulting in a substantial decrease in RyhB stability, a modest reduction in CyaR stability, and no effect on GcvB turnover. The removal of both the KH and S1 domains had a similar effect to deleting S1 domain alone.

We next examined whether the decrease in sRNA stability in the mutants led to a corresponding increase in the expression of negatively regulated mRNA targets. In these experiments, RyhB, CyaR, and GcvB were tested in strains harboring reporter fusions corresponding to their respective targets (*sodB′-′lacZ*, *ompX′-′lacZ, dppA′-′lacZ*, respectively). Expression of each fusion was compared with and without expression of its regulating sRNA in order to differentiate between effects of *pnp* on expression and regulation of the target fusion by the particular sRNA being tested. For GcvB, we compared expression of the *dppA′-′lacZ* fusion in the presence or absence of chromosomal *gcvB,* whereas for the other fusions we compared expression in a strain harboring an empty vector or expressing that sRNA from a *lac-*based promoter on a plasmid. Comparing the ability of the sRNAs to regulate their cognate transcripts in a wild type strain and derived PNPase mutants, we observed a positive correlation between the effects on sRNA stability and function (Fig. 4D–F). Furthermore, for RyhB and CyaR, stability and function positively correlated with binding by PNPase in vitro. These findings support a model in which PNPase binds and stabilizes sRNAs, leading to an increase in steady-state levels, and consequently a reduction in expression of negatively regulated mRNAs.

### PNPase and Hfq together form a ternary complex with an sRNA

Given that both PNPase and Hfq can bind sRNAs, we explored whether the two proteins could also interact with sRNAs jointly or if the interactions are mutually exclusive. EMSA was performed using purified CyaR, RyhB or GcvB, Hfq, and/or PNPase. The mobilities of the sRNAs were retarded with addition of Hfq or PNPase, and addition of both proteins generated a new band with greater mobility shift (Fig. 3B). This indicates formation of a tertiary complex between Hfq, sRNA, and PNPase. Interestingly, the formation of the supershifted species was significantly reduced for the PNPase active site mutant (S437-9A), reflecting either the lower affinity of this mutant for the sRNA, or the important role of these residues in the formation or stability of the ternary complex (Fig. 3B).

### Hfq protects sRNAs from PNPase attack

**FIGURE 5.**
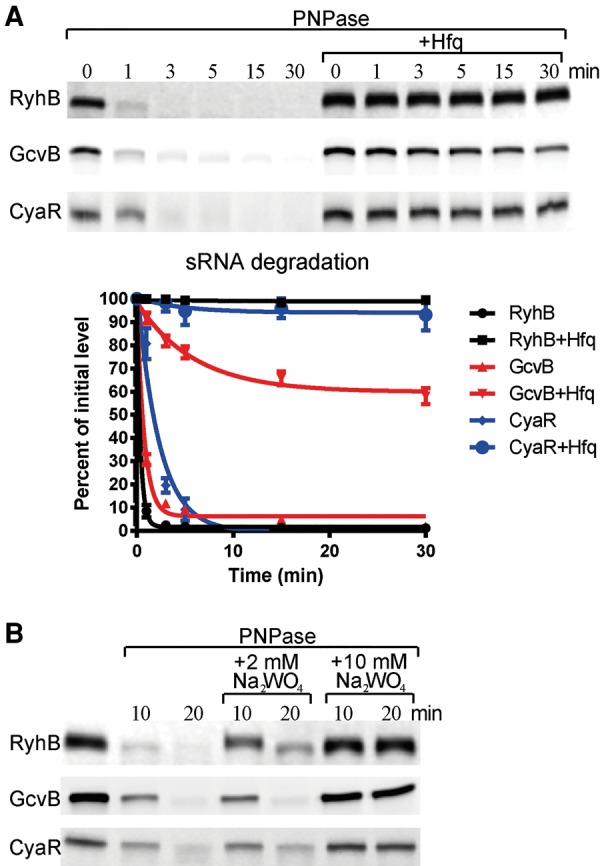
Hfq protects some sRNAs from PNPase degradation. (*A*) In vitro assay on sRNA degradation by PNPase in the presence and absence of Hfq. RyhB, GcvB, and CyaR were incubated with PNPase or PNPase and Hfq in the presence of 2 mM phosphate. Reactions were quantified and standard deviations were estimated from triplicates (*lower* panel). (*B*) In vitro assay of sRNA degradation in the presence of tungstate. Assays were performed as in *A*, except tungstate was added at a final concentration of 2 or 10 mM.

The coIP experiments show that PNPase can stably bind sRNAs without degrading them. However, in vitro PNPase degraded RyhB, GcvB, and CyaR efficiently under catalytic conditions (Fig. 5). The question naturally arises as to how the sRNAs remain protected in vivo. We note that addition of Hfq in 1:1 stoichiometry with sRNA inhibited PNPase degradation (Fig. 5), even though the presence of the chaperone does not interfere with PNPase binding, as shown by the occurrence of the supershifted species in Figure 3B. The stability of the coimmunoprecipitated sRNAs with PNPase described above could be due in part to the copurification with Hfq in a ternary complex. As mentioned, Hfq and PNPase have been reported earlier to copurify (Mohanty et al. 2004; Butland et al. 2005; Obregon et al. 2015), and we observed that Hfq copurified with FLAG-tagged PNPase (Fig. 1C; Supplemental Table S4).

To explore the possibility the ternary complex between Hfq, sRNA, and PNPase could represent the protective complex that ensures sRNA stability, we examined whether PNPase and Hfq protect RyhB from degradation by RNase E (1–762)/RhlB in vitro. The presence of Hfq confers a modest degree of protection from RNase E attack; however, addition of PNPase did not result in further protection (Supplemental Fig. S2). It is possible that there are additional proteins or RNAs present in vivo that are important for PNPase-mediated sRNA protection that are absent from these reactions.

### PNPase improves the binding of MicA sRNA to Hfq in vivo

**FIGURE 6.**
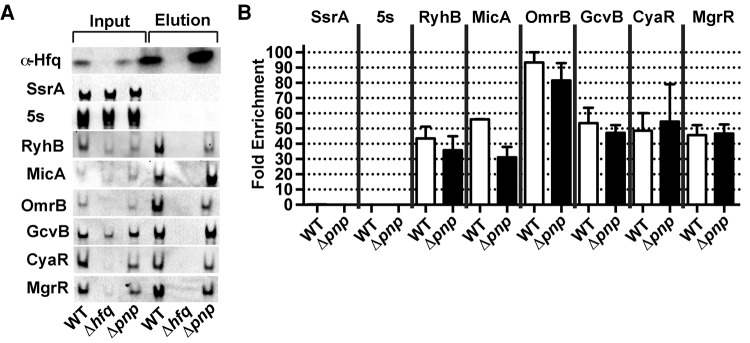
PNPase enhances Hfq-binding of MicA sRNA. (*A*) Northern blot analysis of RNAs coimmunoprecipitated with Hfq. Hfq was immunoprecipitated from extracts of a wild-type strain (KR10000; WT), *hfq* deletion strain (NRD1094; Δ*hfq*), or *pnp* deletion strain (NRD1369; Δ*pnp*) prepared from late exponential phase cultures using anti-Hfq antibody bound to protein A-sepharose resin. For Northern blots, the ratio of the amount of RNA from the input and elution fractions loaded was 16:1, and for Western blots, the ratio of protein samples loaded was 1:18. (*B*) Fold enrichment of a given RNA upon immunoprecipitation with Hfq was calculated as described in Figure 1B.

The results of the EMSA assays indicated that PNPase can form a ternary complex with Hfq and sRNA. To assess the effects of PNPase on Hfq binding of sRNAs in vivo, we immunoprecipated Hfq and then probed for a diverse set of RNAs belonging to different classes according to mode of binding to Hfq (Zhang et al. 2013). Lysates from late exponential phase cultures of wild type, Δ*hfq*, and Δ*pnp* strains were prepared and Hfq immunoprecipitations were performed as previously described (Zhang et al. 2002). We tested several strong class I sRNAs, which bind to Hfq via the proximal face and rim (RyhB, MicA, and OmrB), class II sRNAs which bind the proximal and distal face of Hfq (CyaR and MgrR), and an intermediate class sRNA (GcvB). In these Hfq coimmunoprecipitation experiments, most sRNAs were similarly enriched in the presence or absence of PNPase (Fig. 6); however, we note that the overall levels of RyhB, OmrB, CyaR, and MgrR were significantly lower in the *pnp* deletion strain. In contrast, MicA pulled down less efficiently with Hfq in the absence of PNPase. SsrA and 5S rRNA did not immunoprecipitate with Hfq, as expected.

## DISCUSSION

Studies over several decades have demonstrated that PNPase primarily functions as an exoribonuclease that degrades RNAs processively in a 3′ to 5′ direction (for review, see Andrade et al. 2009); however, PNPase under specific conditions functions “backwards” as a template-independent polynucleotide polymerase in vivo (Mohanty and Kushner 2000). It has more recently been shown that PNPase performs an additional function in *E. coli* to protect Hfq-dependent sRNAs from degradation (De Lay and Gottesman 2011; Andrade et al. 2012). A global analysis of steady-state RNA levels in *E. coli* demonstrated that many RNAs are at higher levels in the absence of PNPase, as might be expected for a degradative role of the enzyme; however, some species including sRNAs are reduced in a *pnp* deletion mutant (Mohanty and Kushner 2003; Pobre and Arraiano 2015), consistent with an RNA stabilizing role. Altogether, these accruing results indicate that PNPase plays multiple functional roles in the cell.

Here, we have shown that PNPase interacts with sRNAs that it protects, and it can do this either in the presence or absence of Hfq, although the former yields more effective binding (Fig. 1). Furthermore, in the absence of PNPase, Hfq does not bind as efficiently at least one sRNA, MicA (Fig. 6). Consistent with previous findings (Mohanty et al. 2004; Butland et al. 2005; Morita et al. 2005; Obregon et al. 2015), PNPase copurifies with Hfq and is likely to form complexes that are at least transient in vivo (Fig. 1C; Supplemental Table S4). In vitro, PNPase binds sRNAs with high affinity and forms a tertiary complex with Hfq and sRNAs (Fig. 3). Within this complex, PNPase is unable to degrade sRNAs (Fig. 5), hinting that the functional consequence of the interaction may be to shelter or remodel the sRNA in vivo.

### PNPase interacts with certain RNAs in a nondestructive mode

All of the Hfq-binding sRNAs that we examined coimmunoprecipitated with PNPase, whereas other very abundant small noncoding RNAs such as 5S rRNA and SsrA did not. Furthermore, when we performed high-throughput sequencing of our inputs and elutions from PNPase coimmunoprecipitations, 11 different Hfq-dependent sRNAs were found to be enriched by at least twofold or more. One limitation of our sequencing approach was that the cDNA library prepared from the RNA was purified with a matrix that preferentially binds DNAs longer than 200 nucleotides (Ampure XP). Therefore, this likely resulted in a reduced number of reads for many of the sRNA detected and prevented other sRNAs from being identified. sRNAs that immunoprecipitated with PNPase showed no discernible degree of degradation, suggesting that PNPase binds these sRNAs in a nondestructive mode. This is not the first example of such a binding mode for this enzyme. Human PNPase, with 40% identity to the *E. coli* homolog, binds the ribozymes RNase P and MRP, as well as 5S RNA and transports them into the mitochondria (Wang et al. 2010), suggesting that PNPase can bind RNAs without degrading them, and binding of the RNA by this enzyme in itself plays an important physiological role. Additionally, in *S. enterica* and *Deinococcus radiodurans*, PNPase binds, but does not degrade Y-RNAs, which are structural RNAs that tether PNPase to the Ro autoantigen homolog Rsr (Chen et al. 2007, 2013). In *D. radiodurans*, Rsr targets rRNA for degradation by PNPase in response to starvation (Wurtmann and Wolin 2010).

Closer examination of how PNPase interacts with Hfq-dependent sRNAs in vitro revealed that the S1 domain is important for binding RyhB, CyaR, and GcvB (Fig. 3). Substitution of the active site serines predicted to bind the phosphate used for nucleophilic attack slightly reduced sRNA binding. In vivo binding studies were less clear. For RyhB, both the S1 domain and active site residues appeared to be important for binding, but for GcvB and CyaR a substantial difference in sRNA binding between the wild-type PNPase, S1 domain mutant, or active site mutant was not observed. It is worth noting that the deletion of the S1 domain of PNPase also had much more of an effect on the stability and function for RyhB than for GcvB or CyaR (Fig. 4). The apparent discrepancy between the in vitro and in vivo results in terms of PNPase residues important for sRNA binding may be due to differences in RNA concentration. In the EMSA assays, the RNA concentration used corresponds to ∼200 molecules per cell, whereas GcvB and CyaR levels may be higher in the cells harvested for the coimmunoprecipitation experiment, overriding the requirements for S1 domain binding. This is consistent with previous findings, demonstrating that PNPase can bind RNA in the absence of the S1 domain at high RNA concentrations (Stickney et al. 2005). All together, these results suggest that the active site and S1 domains contribute to sRNA binding, but are not the sole determinants in vivo.

### PNPase forms a ternary complex with Hfq and sRNA

Our results support a ternary complex comprised of PNPase, Hfq, and sRNA (Figs. 1C, 3; Supplemental Table S4) and suggest that PNPase and Hfq may also form a ribonucleoprotein complex in the cell. In our in vitro studies, PNPase readily degrades sRNAs in the absence of Hfq (Fig. 5), but binds and is unable to degrade sRNAs in its presence (Figs 3, 5). In our PNPase immunoprecipitation experiments, sRNAs pulled down less efficiently in an *hfq* null mutant (Fig. 1). At least one sRNA, namely MicA, is less efficiently coimmunoprecipitated with Hfq in the absence of PNPase, also supporting the existence of a ternary complex. Other sRNAs such as RyhB, OmrB, CyaR, GcvB, and MgrR showed the same fold enrichment upon Hfq coimmunoprecipitation in the presence or absence of PNPase, but the overall levels of these sRNAs were much lower in the *pnp* deletion mutant. Since Hfq protects these sRNAs from degradation, the reduced levels of these sRNA may indicate reduced Hfq binding. Alternatively, these results could indicate that in the absence of PNPase, sRNAs bind to Hfq in a manner that is not fully protective from RNases. These findings naturally raise the question as to how PNPase would interact with sRNAs in an Hfq–PNPase–sRNA complex. Hfq interacts with the poly(U) tail of Rho-independent terminators of sRNAs (Otaka et al. 2011; Sauer and Weichenrieder 2011), and proximal site residues involved in binding the poly(U) tail form critical interactions with all Hfq-binding sRNAs tested (Zhang et al. 2013). Hfq also interacts with other sequences in sRNAs via additional residues. For class I sRNAs, an arginine patch along the rim of Hfq interacts with an AU-rich sequence upstream of a stem–loop structure, and for class II sRNAs, Hfq can interact with ARN repeats via distal face residues (Zhang et al. 2013; Schu et al. 2015). One possibility is that PNPase accesses the 3′ end of the Hfq bound sRNA, begins degrading the sRNA in the 3′ to 5′ direction, and remains stalled at the 3′ end forming a protective Hfq–PNPase–sRNA complex. These removed nucleotides could be template encoded or ones added by poly(A) polymerase. Alternatively, it is possible that PNPase binds to a site on the Hfq-bound sRNA other than the 3′ end, and the 3′ end remains associated with Hfq or is otherwise remodeled and inaccessible to the active site of PNPase. Rsr, mentioned earlier, forms a structure of similar shape and molecular weight as the Hfq hexamer and interacts with PNPase via a small noncoding Y-RNA that is not degraded by PNPase. Internal ribonucleotide sequences located within two stem–loop structures of the Y-RNA are important for PNPase binding (Chen et al. 2014). Given the apparent similarities between these systems, we anticipate that the RsrR-Y-RNA-PNPase (Chen et al. 2013) and Hfq-sRNA-PNPase complexes may form analogous structures. We envisage that PNPase contributes in a direct and complex way to sRNA actions in the cell by influencing in a dynamic manner the composition of sRNAs that will be presented on Hfq and possibly other regulatory ribonucleoprotein complexes.

## MATERIALS AND METHODS

### Bacterial strains and plasmids

All strains described in this study are derivatives of *E. coli* K-12 strain MG1655 (Supplemental Table S1). Plasmids used in this study are also listed in Supplemental Table S1. Primers and 5′ biotinylated oligonucleotide probes were purchased from Integrated DNA Technologies, Inc. or Sigma-Aldrich Co., LLC (Supplemental Table S2). Strain construction is described in the Supplemental Material.

### Culture media and growth conditions

*Escherichia coli* strains were grown in Lennox broth liquid medium or agar plates. Antibiotics were used at the following concentrations: ampicillin, 100 mg L^−1^; chloramphenicol, 10 mg L^−1^; kanamycin, 25 mg L^−1^; rifampicin, 500 mg L^−1^; and tetracycline, 25 mg L^−1^. 2,2′ dipyridyl (dipyridyl), isopropryl β-d-1-thiogalactopyranoside (IPTG) and arabinose was used at a final concentration of 250 μM, 100 μM, and 0.01%, respectively.

### β-Galactosidase assays

To measure expression of OmpX, overnight cultures of strains expressing an *ompX′-′lacZ* translational fusions harboring pBR-plac or pNRD405 were diluted 200-fold in LB medium containing ampicillin and grown to an OD_600_ between 0.3 and 0.4. A sample was then taken, and β-galactosidase assays were performed as described by Miller (1992). SodB expression was measured by a similar approach using strains harboring a *sodB′–′lacZ* translation fusion and pBR-plac and pBR-plac-RyhB. DppA expression was measured via a *dppA′–′lacZ* translational fusion in a *gcvB*^*+*^ or Δ*gcvB* strains.

### Northern blotting

To determine the effects of mutation in *pnp* on RyhB stability, the wild-type strain or derived *pnp* mutant was grown to exponential phase (OD_600_ of 0.3 to 0.4) at 37°C. RyhB was induced for 15 min with dipyridyl and then a 700-μL sample was removed from each culture. Sixteen minutes after induction, rifampicin was added and additional samples were removed. Total RNA was extracted using the hot phenol method as previously described (Masse et al. 2003). These samples were also used to examine GcvB turnover as GcvB is highly expressed under these growth conditions. For CyaR, the experiment was performed similar to RyhB except strains had *cyaR* deleted from the chromosome and harbored a plasmid encoded *cyaR* that is transcribed from a *lac*-based promoter. Thus, ampicillin was added to the cultures to maintain the plasmid, and the sRNA was induced with IPTG instead of dipyridyl.

After purification by the hot phenol method, 2 μg of RNA was fractionated on a 10% Tris-borate-EDTA (TBE)-urea polyacrylamide gel in 1× TBE at 55 V for 3 h. Prior to the addition of RNA samples, the TBE-urea gel was run for 30 min at 55 V. The total fractionated RNA was transferred to a Zeta-Probe GT membrane (Bio-Rad) by electroblotting using the Transblot SD semi-dry transfer cell at 400 mA for 45 min and UV-crosslinked to the membrane. After prehybridization of the membrane in ULTRAhyb solution (Ambion) at 42°C, the appropriate 5′-biotinylated probe was added to a final concentration of 100 ng per mL, and the membrane was incubated with probe overnight. The blot was developed according to the procedure of the Brightstar Biodetect kit (Ambion) and Chemidoc MP imager (Bio-Rad). RNA concentration was quantified with the Nanodrop 2000 (Thermo Scientific). Signal intensity for blots was quantified with the Bio-Rad Image Lab software.

### Immunoprecipitation of PNPase

Overnight cultures were diluted 200-fold into fresh LB medium and cultured to an OD_600_ of 1.0. For immunoprecipitations described in Figure 1C, in vivo formaldehyde crosslinking was performed as previously described (Vakulskas et al. 2014). 25 mL aliquots of cells were pelleted at 6000*g* for 10 min at 4°C. Each aliquot of cells was washed twice with 1.0 mL of TBS buffer pH of 7.4 (50 mM Tris, 150 mM NaCl) with or with tungstate (2 mM) and pelleted after each wash at 18,000*g* for 5 min at 4°C. Cells were suspended in 400 μL of TBS buffer with or without tungstate containing 4 μL of HALT protease inhibitor (Thermo Scientific) and 2 μL of Superase (Ambion), added to 400 μL of glass beads (0.1 mm), and disrupted at 4°C for 10 min using the Disruptor Genie. 700 μL of TBS buffer was added followed by vortexing for 30 sec. Cell debris was removed by centrifugation at 18,000*g* for 30 min at 4°C. The soluble lysate was placed in a new tube and the volume made up to 1 mL. A 50-μL sample was reserved for Northern or Western blotting (input). Superase was then added to the rest of the sample, which was subsequently added to 60 μL of anti-FLAG M2 agarose (Sigma) that was washed in TBS buffer per manufacturers’ instructions for batch purification. The cell lysate was incubated while mixing at 4°C for 2 h. The resin was washed three times with 1.5 mL of TBS buffer with or without tungstate, and eluted for 30 min at 4°C with 500 μL of TBS buffer with 3xFLAG peptide (Sigma). 25 μL of elution was reserved for Western blot analysis. RNA from the remaining sample and initial input RNA sample that was reserved was extracted by the hot phenol lysis method. Northern blot was performed as described above. Axeq Technologies performed the RNA sequencing, alignment of reads using Bowtie, and expression analysis as previously described (Mortazavi et al. 2008). RNA samples were treated with DNase Turbo (Ambion) prior to sequencing.

Western blotting analysis was performed by fractionating the samples reserved for protein analysis on a NuPAGE 4%–12% polyacrylamide gel at 180 V for 1 h in NuPAGE MOPS SDS running buffer (Life Technologies, Inc.) The fractionated protein was transferred to a 0.2 μM PVDF membrane using the Transblot SD semi-dry blotter at 15 V for 30 min. The membrane was blocked with PBST of pH 7.4 (11.9 mM phosphate, 137 mM NaCl, 2.7 mM KCl, 0.1% Tween 20) containing nonfat milk (3%) overnight, washed three times with PBST, probed with a rat anti-FLAG antibody (1:1000) for 1 h, washed three times with PBST, incubated with anti-rat goat antibody conjugated to alkaline phosphatase (1:2500), and washed three additional times with PBST. For detection of the β- and β′-subunits of RNA polymerase anti-β (8RB13) and anti-β′ (NT73) mouse monoclonal antibodies and anti-mouse goat antibody conjugated to alkaline phosphatase were used per manufactures’ instructions (Santa Cruz Biotechnologies, Inc). The membrane was then rinsed well with Millipore purified water. The secondary conjugated antibody was then visualized using the Lumi-Phos WB chemiluminescent substrate and the Chemidoc MP imager (Bio-Rad). Signal intensity was quantified with the Bio-Rad Image Lab software.

### Immunoprecipitation of Hfq

Overnight cultures were diluted 200-fold into 25 mL of fresh LB liquid medium and grown to an OD_600_ of 1.0. Cells were pelleted, washed, and frozen as described above for the PNPase immunoprecipitation. Immunoprecipitations were performed as previously described (Zhang et al. 2002) using anti-Hfq antiserum obtained from Dr. Susan Gottesman (NCI). RNA was isolated by phenol extraction as described above. Protein for Western blot analysis was precipitated from the phenol fraction from the RNA extraction with 2 volumes of cold acetone and pelleted at 18,000*g* for 30 min. The pellet was washed twice with 1 volume of cold acetone, pelleted at 18,000*g* for 10 min after each wash, and was allowed to air dry. The protein was then suspended in Laemmli sample buffer. Western blots were performed as described above, except using anti-Hfq antiserum and goat anti-rabbit IgG secondary antibody conjugated to alkaline phosphatase (Thermo Fisher Scientific).

### PNPase purification

PNPase was expressed from pET-Duet vector in BL21(DE3) cells induced with 0.5 mM IPTG at 25°C. Cells were harvested by centrifugation 3–4 h after induction and suspended in lysis buffer (20 mM Tris-HCl, pH 8.0, 150 mM NaCl, 150 mM KCl, 5 mM MgCl_2_, EDTA-free protease inhibitor cocktail [Roche]). After lysis, the lysate was clarified by centrifugation (4°C, 30 min, 37,500*g*) and PNPase was precipitated with ammonium sulfate (51.3% saturation) for 1 h at 4°C. The sample was centrifuged (4°C, 30 min, 37,500*g*), and the pellet was suspended in Q Buffer A (20 mM Tris-HCl, pH 8.5, 30 mM NaCl, 1 mM DTT, 10% v/v glycerol, EDTA-free protease inhibitor cocktail [Roche]), loaded on 5 mL HiTrap Q column (GE Healthcare) and eluted with a gradient of Q Buffer B (Q Buffer A with 1 M NaCl). Fractions from Q column were evaluated by SDS-PAGE and those containing PNPase were pooled and supplemented with MgCl_2_ (1 mM total concentration), sodium phosphate pH 7.9 (50 mM total concentration), (NH_4_)_2_SO_4_ (0.9 M total concentration) and DTT (1 mM total concentration). The protein solution was then loaded on a 5 mL HiTrap butyl-sepharose column (GE Healthcare) equilibrated with BS Buffer A (50 mM Tris-HCl, pH 7.9, 1 M (NH_4_)_2_SO_4_, EDTA-free protease inhibitor cocktail [Roche]) and eluted with a gradient of BS Buffer B (50 mM Tris-HCl, pH 7.9, EDTA-free protease inhibitor cocktail [Roche]). Fractions containing PNPase were pooled, concentrated, and loaded on a Superdex 200 16/60 gel filtration column equilibrated with a buffer composed of 20 mM Tris-HCl, pH 8.0, 150 mM NaCl, 5 mM MgCl_2_, 5% v/v glycerol, 1 mM DTT, and EDTA-free protease inhibitor cocktail (Roche).

### In vitro transcription

RNA was obtained by in vitro transcription according to standard protocols, using purified PCR product as a template (for primer sequences, see Supplemental Table S2). Two forms of RyhB were used in this study that differed by the length of the polyU tail: RyhB with 9 Us on 3′ end was used except in the ITC and EMSA experiments in the presence of tungstate, where RNA with 4Us on the 3′ end was used.

### RNA degradation assays

Degradation assays were performed in buffer containing 20 mM Tris, pH 7.5, 100 mM NaCl, 1 mM MgCl_2_, 1 mM DTT, 2 mM sodium phosphate. Degradation assays were carried out at 37°C. 0.2 µM RNA and 0.2 µM PNPase were used per reaction. RNA was heated for 2 min at 50°C and slowly cooled to room temperature. PNPase and Hfq were preincubated together before addition of RNA. Reactions were stopped by incubation with Proteinase K in 50°C for 15–30 min, in Proteinase K buffer (100 mM Tris-HCl, pH 7.5, 12.5 mM EDTA, 150 mM NaCl, 1% wt/v SDS). An initial time point was taken from the reaction mixture immediately after the RNA was added and corresponds to 10–15 sec. RNA loading dye (Fermentas) was added and after denaturation at 95°C for 3 min whole samples were loaded on a 10% polyacrylamide gel containing 7 M urea. Gels were stained in SYBR Gold solution (Invitrogen).

### Gel mobility shift assays

Binding experiments were carried out at 30°C. 0.2 µM RNA was used per reaction. Gel-shift assays were performed in buffer containing 20 mM Tris, pH 7.5, 100 mM NaCl, 1 mM MgCl_2_, 1 mM DTT. After 20 min from addition of RNA, samples were transferred to ice, supplemented with 5 µL of 50% glycerol in binding buffer and immediately loaded on the native gel.

### Isothermal titration calorimetry

PNPase and RyhB were buffer exchanged in 20 mM Tris-HCl, pH 7.5, 1 mM MgCl_2_, 1 mM DTT using Micro Biospin 6 Chromatography Columns (Bio-Rad). Measurements were performed on a Microcal ITC200 calorimeter at 30°C with stirring at 1000 rpm. Following an initial injection of 0.4 μL, injections of 2 μL RyhB (27 μM syringe concentration) into PNPase (2.7 μM cell concentration) were separated by 100 sec to allow the system to reach equilibrium between injections. Data were analyzed using the ORIGIN software.

### Bio-layer interferometry

Kinetic measurements were performed using Octet RED96 system (ForteBio, UK). RyhB was in vitro transcribed in the presence of fivefold excess of GMP-biotin (TriLink Biotechnologies, San Diego CA, USA) over GTP. Biotin-labeled RNA was immobilized on the Streptavidin biosensors and subsequently submerged into 5 µM solution of maltose-binding protein (MBP) labeled with biotin. Binding was performed in the binding buffer 20 mM Tris, pH 7.5, 100 mM NaCl, 1 mM MgCl_2_, 1 mM DTT supplemented with 1 mg/mL acetylated BSA (Affymetrix). Sensors were regenerated between measurements with 1 M MgCl_2_. Another set of tips was saturated with MBP-biotin and used for determination of nonspecific binding. The data were fitted with Data Analysis software (ForteBio) with a 1:1 binding model. The plots were prepared with Profit (Quantum Soft, Switzerland) using the following equation for response fit:

$$Y=\mathrm{Rm}\times X^{n}/(K_{d}^{\;\;n}\;+\;X^{n}),$$

where *Y* is the observed binding, *X* is the molar concentration of the ligand, Rm is the maximum specific binding, and *n* is the Hill's coefficient.

## DATA DEPOSITION

The high-throughput RNA sequencing data presented in this publication have been deposited in NCBI's Gene Expression Omnibus (GEO) under accession number GSE69856 and will be released to the public upon publication of this manuscript.

## SUPPLEMENTAL MATERIAL

Supplemental material is available for this article.

## Supplementary Material

Supplemental Material
