# Supplementary material for: The ribonuclease polynucleotide phosphorylase can interact with small regulatory RNAs in both protective and degradative modes
Source: RNA. 2016 Mar;22(3):360–72. doi: 10.1261/rna.052886.115 (PMC4748814; doi:10.1261/rna.052886.115)
Supplement: Supplemental Material [file supp_22_3_360__index.html]

The ribonuclease polynucleotide phosphorylase can interact with small regulatory RNAs in both protective and degradative modes — The ribonuclease polynucleotide phosphorylase can interact with small regulatory RNAs in both protective and degradative modes — Supplemental Material 

# The ribonuclease polynucleotide phosphorylase can interact with small regulatory RNAs in both protective and degradative modes

## Supplemental Material

**Files in this Data Supplement:**

- Supp Table S3.xlsx
- Supp Material.docx
