# Supplementary material for: The ribonuclease polynucleotide phosphorylase can interact with small regulatory RNAs in both protective and degradative modes
Source: RNA. 2016 Mar;22(3):360–72. doi: 10.1261/rna.052886.115 (PMC4748814; doi:10.1261/rna.052886.115)
Supplement: Supplemental Material [file supp_052886.115_SuppMaterial.docx]

**SUPPLEMENTARY INFORMATION**

**The exoribonuclease polynucleotide phosphorylase can interact with small noncoding RNAs in both protective and degradative modes.**

Katarzyna J. Bandyra^1*^, Dhriti Sinha^2^, Johanna Syrjanen^1^, Ben F. Luisi^1^, and Nicholas R. De Lay^2,3*✝^

^1^Department of Biochemistry, University of Cambridge, Tennis Court Road, Cambridge CB2 1GA, U. K.

^2^Department of Microbiology and Molecular Genetics, University of Texas Medical School, Houston, TX 77030, USA

^3^Graduate School of Biomedical Sciences, University of Texas Health Science Center, Houston, TX 77030

^*^ K.J.B. and N.R.D contributed equally to this work.

^✝^ Correspondence should be sent to Nicholas R. De Lay. Tel: +1 (713) 500-6293; Email: nicholas.r.delay@uth.tmc.edu

**TABLE OF CONTENTS**

**Figure S1: Activity of PNPase-FLAG in processing RNA and facilitating sRNA-mediated regulation of gene expression.**

**Figure S2: PNPase does not protect sRNAs from degradation by RNase E in vitro.**

**Table S1: Strains and plasmids used in this study**

**Table S2: Primers and probes used in this study**

**Table S3: RNAs co-immunoprecipitated with PNPase in the presence or absence of tungstate.**

**Table S4: Proteomics analysis of immunoprecipitated Hfq and PNPase**

**Supplementary Materials and Methods**

**Supplementary References**


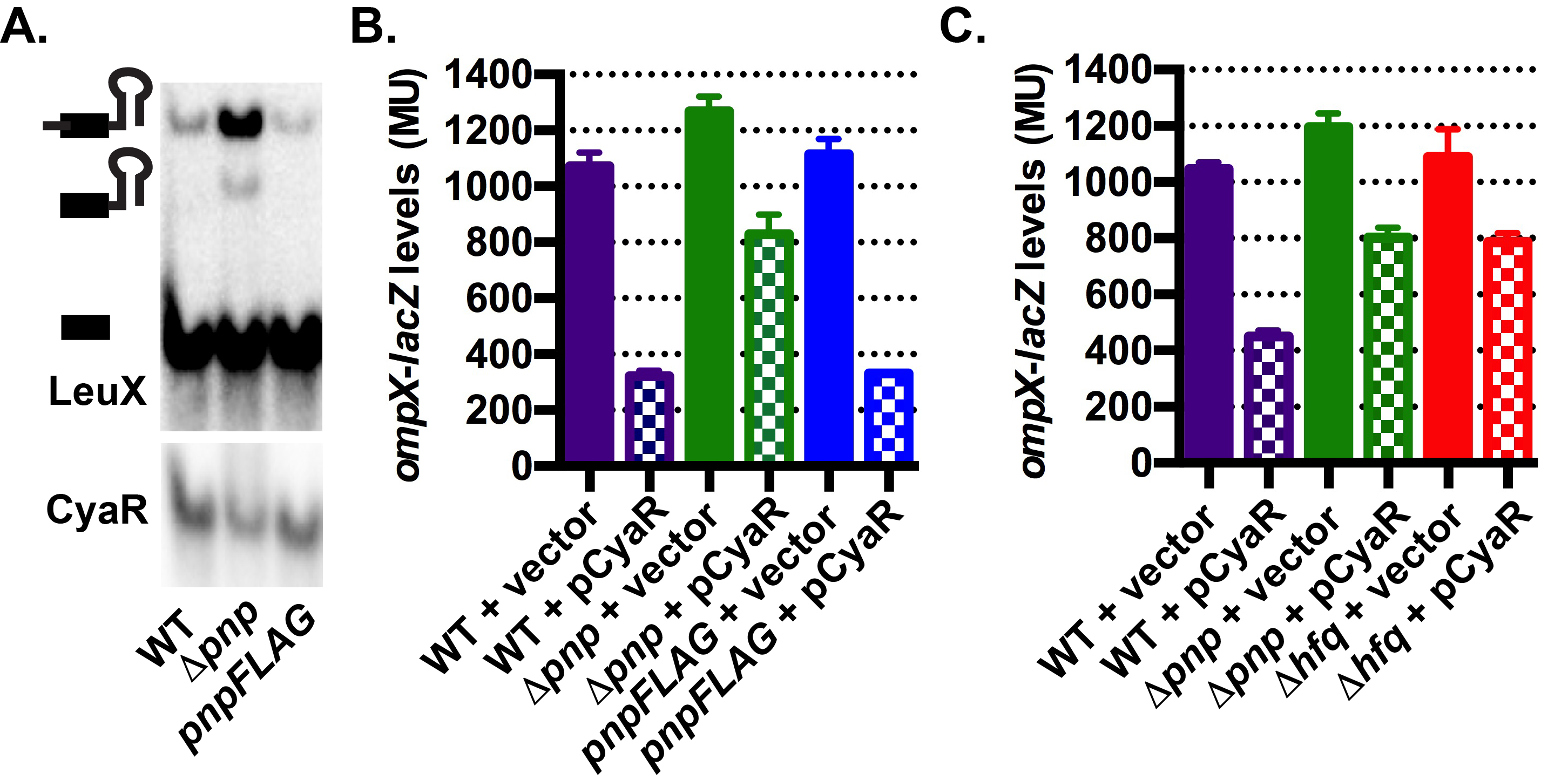


**Figure S1: Activity of PNPase-FLAG in processing RNA and facilitating sRNA-mediated regulation of gene expression.** (A) LeuX processing and CyaR levels were analyzed by northern blot. RNA was isolated from late exponential phase cultures (OD600 ~1.0) of a wild type (KR10000; WT) or derived Δ*pnp* (NRD999) or *pnpFLAG* strain (NRD1243), fractionated on a polyacrylamide gel, transferred to a nylon membrane, and probed for LeuX tRNA or CyaR sRNA. Deletion of *pnp* led to a nearly 500% increase in the unprocessed transcript compared to the wild type strain, but the deletion did not significantly affect the total amount of mature LeuX transcript generated, consistent with previous findings (Mohanty and Kushner 2010). (B) Response of *ompX*-*lacZ* expression to CyaR. Samples were analysed for β-galactosidase activity from exponential phase cultures (OD600 ~0.3) of a *cyaR* deletion strain containing an *ompX-lacZ* translational fusion (NRD377; WT), a derived strain that has *pnp* replaced with an antibiotic resistance cassette (NRD677; Δ*pnp;*) or 3XFLAG-tagged PNPase encoding allele (NRD1267; *pnpFLAG*) and harboring a vector (pBR-plac) or a plasmid expressing CyaR (pNRD405). Deletion of *pnp* resulted in a defect in the regulation of the *ompX*’-‘*lacZ* fusion by CyaR, as previously reported (De Lay and Gottesman 2011). (C). β-galactosidase activity assays as described in (B) with a wild type (WT; NRD726), or derived Δ*pnp* (NRD730) or Δ*hfq* strain (NRD731).

**
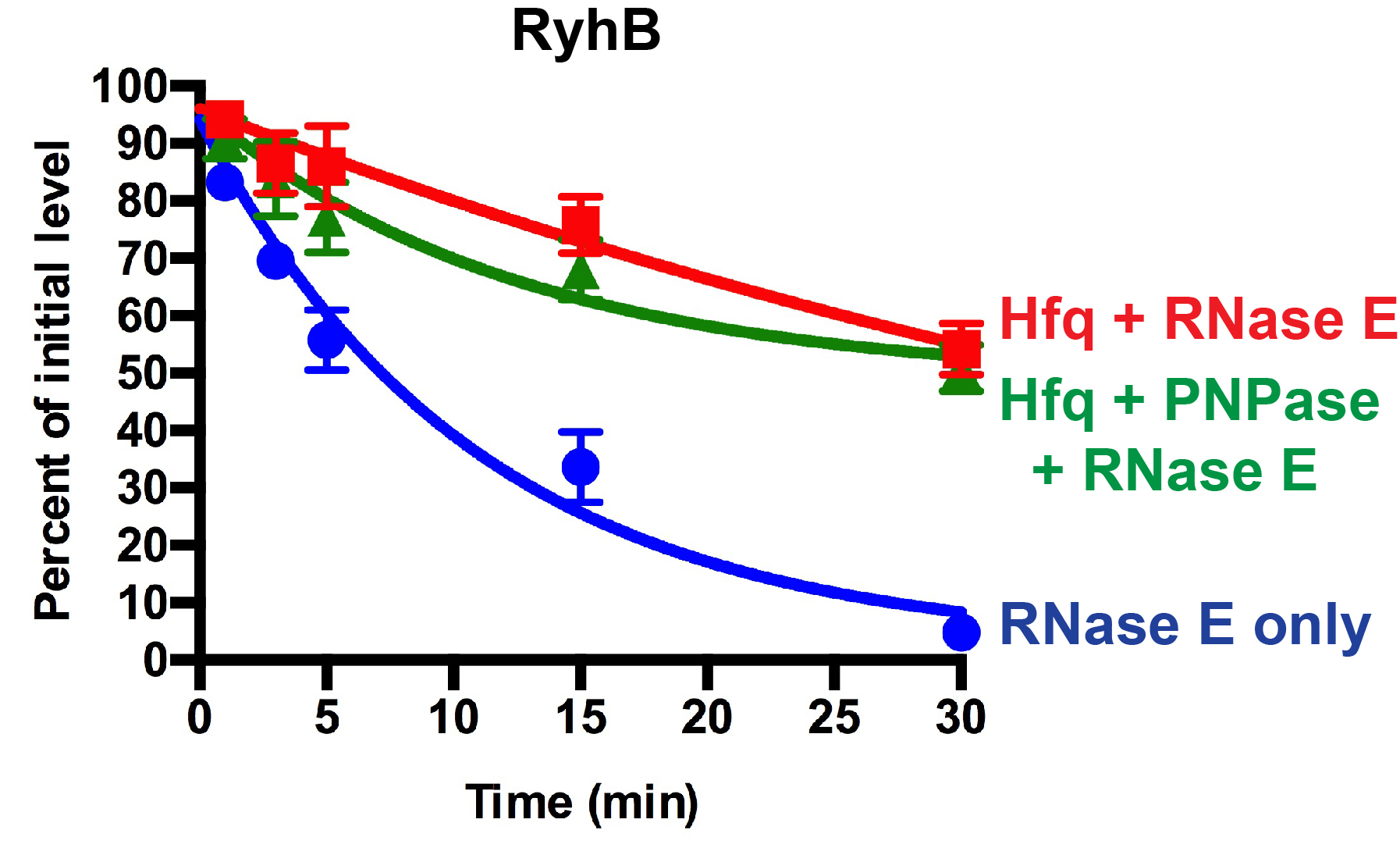
**

**Figure S2: PNPase does not protect sRNAs from degradation by RNase E in vitro.**

Degradation of RyhB by RNase E. Hfq confers some protection against RNase E attack, but this protective effect is not significantly increased by the presence of PNPase. The reaction conditions do not include phosphate and therefore would not support phosphorylysis by PNPase.

**Table S1: Strains and plasmids used in this study**

| **Strain or plasmid** | **Relevant features** | **Origin** |
| --- | --- | --- |
| **Strains** |  |  |
| CR201 | *cya::kan ccdB* | C. Ranquet |
| EM1377 | MG1655 Δ*lacX74 rne-131 zce-726*::Tn*10* | (Masse et al. 2003) |
| KM86 | *mal*::*lacI^q^ ΔaraBAD lacI’-*P_BAD_*::dppA’-’lacZ* | (Moon and Gottesman 2011) |
| KR10000 | MG1655 rph^+^ | Donald Court, NCI |
| JW3140 | Δ*αrgG*::*kan* | KEIO Collection (Baba et al. 2006) |
| MG1267 | Δ*micC*::*kan* | Susan Gottesman, NCI |
| NM580 | MG1655 lacI-T1T2-*zeo^R^*-pBRpLacO-kan-pBAD-ccdB. Mini-λ-Red::*tet^R^* | (Battesti et al. 2015) |
| NM1200 | MG1655 mini-λ cm^r^ | N. Majdalani, NCI |
| NM22565 | Δ*hfq*::*cat sacB* | N. Majdalani, NCI |
| NRD377 | MG1655 Δ*lacX74* *mal*::*lacI*^q^ P_BAD_::*ompX*::*lacZ* Δ*cyaR*::*cat* | (De Lay and Gottesman 2009) |
| NRD463 | MG1655 Δ*pnp*::*cat* | (De Lay and Gottesman 2011) |
| NRD465 | MG1655 Δ*pnp*::*kan* | (De Lay and Gottesman 2011) |
| NRD473 | MG1655 Δ*lacX74* *mal*::*lacI*^q^ Δ*pnp*::*kan* | (De Lay and Gottesman 2011) |
| NRD537 | *mal*::*lacI^q^ ΔaraBAD lacI’-*P_BAD_*::sodB’-’lacZ* | This study |
| NRD576 | MG1655 Δ*lacX74* *mal*::*lacI*^q^ Δ*pnp*::*kan rne-131 zce-726*::Tn*10* | NRD473 + P1(EM1377) |
| NRD677 | MG1655 Δ*lacX74* *mal*::*lacI*^q^ P_BAD_::*ompX*::*lacZ* Δ*cyaR*::*cat* Δ*pnp*::*kan* | (De Lay and Gottesman 2011) |
| NRD726 | MG1655 Δ*lacX74* *mal*::*lacI*^q^ P_BAD_::*ompX*::*lacZ* Δ*cyaR* | *cat* cassette removed by FLP (pCP20) |
| NRD730 | MG1655 Δ*lacX74* *mal*::*lacI*^q^ P_BAD_::*ompX*::*lacZ* Δ*cyaR* Δ*pnp*::*kan* | NRD726 + P1(NRD465) |
| NRD731 | MG1655 Δ*lacX74* *mal*::*lacI*^q^ P_BAD_::*ompX*::*lacZ* Δ*cyaR* Δ*hfq*::*cat sacB* | NRD726 + P1(NM22565) |
| NRD999 | MG1655 rph^+^ Δ*pnp*::*cat* | KR10000 + P1(NRD463) |
| NRD1009 | MG1655 rph^+^ Δ*pnp::kan ccdB* | This study |
| NRD1021 | MG1655 rph^+^ *pnp E550TAA* | This study |
| NRD1022 | MG1655 rph^+^ *pnp S437A S438A S439A* | This study |
| NRD1033 | MG1655 Δ*lacX74* *mal*::*lacI*^q^ P_BAD_::*ompX*::*lacZ* Δ*cyaR*::*cat* Δ*αrgG*::*kan* | NRD377 + P1(JW3140) |
| NRD1044 | MG1655 Δ*lacX74* *mal*::*lacI*^q^ P_BAD_::*ompX*::*lacZ* Δ*cyaR*::*cat pnp E550TAA* | NRD1033 + P1(NRD1021) |
| NRD1045 | MG1655 Δ*lacX74* *mal*::*lacI*^q^ P_BAD_::*ompX*::*lacZ* Δ*cyaR*::*cat pnp S437A S438A S439A* | NRD1033 + P1(NRD1022) |
| NRD1094 | MG1655 rph^+^ Δ*hfq*::*cat sacB* | KR10000 + P1(NM22565) |
| NRD1113 | *mal*::*lacI^q^ ΔaraBAD lacI’-*P_BAD_*::sodB’-’lacZ* Δ*αrgG*::*kan* | NRD537 + P1(JW3140) |
| NRD1114 | *mal*::*lacI^q^ ΔaraBAD lacI’-*P_BAD_*::sodB’-’lacZ* Δ*pnp*::*kan* | NRD537 + P1(NRD576) |
| NRD1116 | *mal*::*lacI^q^ ΔaraBAD lacI’-*P_BAD_*::sodB’-’lacZ pnp E550TAA* | NRD1113 + P1(NRD1021) |
| NRD1117 | *mal*::*lacI^q^ ΔaraBAD lacI’-*P_BAD_*::sodB’-’lacZ pnp S437A S438A S439A* | NRD1113 + P1(NRD1022) |
| NRD1144 | MG1655 rph^+^ *pnp K606TAA* | This study |
| NRD1147 | MG1655 Δ*lacX74* *mal*::*lacI*^q^ P_BAD_::*ompX*::*lacZ* Δ*cyaR*::*cat pnp K606TAA* | NRD1033 + P1(NRD1144) |
| NRD1151 | *mal*::*lacI^q^ ΔaraBAD lacI’-*P_BAD_*::sodB’-’lacZ pnp K606TAA* | NRD1113 + P1(NRD1144) |
| NRD1229 | MG1655 lacI-T1T2-*zeo^R^*- *lacI’-*P_BAD_*::dppA’-’lacZ* | This study |
| NRD1243 | KR10000 *pnp::3XFLAG* | This study |
| NRD1246 | KR10000 *pnpΔS1::3XFLAG* | This study |
| NRD1247 | KR10000 *pnp(S437A S438A S439A)::3XFLAG* | This study |
| NRD1249 | KR10000 *pnp(S437A S438A S439A)::3XFLAG pnp::3XFLAG* | This study |
| NRD1267 | MG1655 Δ*lacX74* *mal*::*lacI*^q^ P_BAD_::*ompX*::*lacZ* Δ*cyaR*::*cat* | NRD1033 + P1(NRD1243) |
| NRD1268 | KR10000 *pnp::ccdB kan::3XFLAG* | This study |
| NRD1269 | KR10000 *pnp(S437A S438A S439A* Δ*S1)::ccdB kan 3XFLAG* | This study |
| NRD1297 | KR10000 *pnp::3XFLAG* Δ*hfq*::*cat sacB* | NRD1243 + P1(NM22565) |
| NRD1316 | MG1655 lacI-T1T2-*cat*- *lacI’-*P_BAD_*::dppA’-’lacZ* | This study |
| NRD1369 | MG1655 rph^+^ Δ*pnp* | *cat* cassette removed by FLP (pCP20) |
| NRD1382 | *mal*::*lacI^q^ ΔaraBAD* lacI-T1T2-*cat*- *lacI’-*P_BAD_*::dppA’-’lacZ* | NRD537 + P1(NRD1316) |
| NRD1383 | *mal*::*lacI^q^ ΔaraBAD* lacI-T1T2-*cat*- *lacI’-*P_BAD_*::dppA’-’lacZ* Δ*pnp*::*kan* | NRD1114 + P1(NRD1316) |
| NRD1384 | *mal*::*lacI^q^ ΔaraBAD* lacI-T1T2-*cat*- *lacI’-*P_BAD_*::dppA’-’lacZ*  *pnp E550TAA* | NRD1116 + P1(NRD1316) |
| NRD1385 | *mal*::*lacI^q^ ΔaraBAD* lacI-T1T2-*cat*- *lacI’-*P_BAD_*::dppA’-’lacZ pnp S437A S438A S439A* | NRD1117 + P1(NRD1316) |
| NRD1387 | KR10000 Δ*gcvB::zeo^R^* |  |
| NRD1388 | *mal*::*lacI^q^ ΔaraBAD* lacI-T1T2-*cat*- *lacI’-*P_BAD_*::dppA’-’lacZ* Δ*gcvB::zeo^R^* | NRD1382 + P1(NRD1387) |
| NRD1389 | *mal*::*lacI^q^ ΔaraBAD* lacI-T1T2-*cat*- *lacI’-*P_BAD_*::dppA’-’lacZ* Δ*pnp*::*kan* Δ*gcvB::zeo^R^* | NRD1383 + P1(NRD1387) |
| NRD1390 | *mal*::*lacI^q^ ΔaraBAD* lacI-T1T2-*cat*- *lacI’-*P_BAD_*::dppA’-’lacZ*  *pnp E550TAA* Δ*gcvB::zeo^R^* | NRD1384 + P1(NRD1387) |
| NRD1391 | *mal*::*lacI^q^ ΔaraBAD* lacI-T1T2-*cat*- *lacI’-*P_BAD_*::dppA’-’lacZ pnp S437A S438A S439A* Δ*gcvB::zeo^R^* | NRD1385 + P1(NRD1387) |
| PM1205 | *mal*::*lacI^q^ ΔaraBAD lacI’-*P_BAD_*::cat sacB::lacZ* | (Mandin and Gottesman 2009) |
|  |  |  |
| **Plasmids** |  |  |
| pBR-plac | Amp^r^; *lac* promoter-based expression vector | (Guillier and Gottesman 2006) |
| pBR-plac-MicC | Amp^r^; expresses MicC from a *lac* promoter | (Mandin and Gottesman 2010) |
| pBR-plac-RyhB | Amp^r^; expresses RyhB from a *lac* promoter | (Mandin and Gottesman 2010) |
| pCP20 | Amp^r^, Cm^r^; λcI857 P_R_*::flp* pSC101 *oriTS* | (Datsenko and Wanner 2000) |
| pNRD405 | Amp^r^; expresses CyaR from a *lac* promoter | (De Lay and Gottesman 2009) |
| pNRD412 | Amp^r^; expresses *pnp* from an *araBAD* promoter | (De Lay and Gottesman 2011) |
| pNRD466 | Ampr; expresses *pnp*S437A S438A S439A and is derived from pNRD405 | This study |
|  |  |  |
|  |  |  |

**Table S2: Primers and Northern blot probes used in this study**

| **Primer or probe** | **Sequence (5’ to 3’)** |
| --- | --- |
| **Primers** |  |
| cat-P1 | TTTCGTTTTATCTGTTTTGAAAGAAGGCCATCCTGACGGATGGCCTTTTTTGTAGGCTGGAGCTGCTTCG |
| cat-P2 | ATTGGTTTCTTCTCTGAATGGTGGGAGTATGAAAAGTATGGCTGAAGCGCATGGGAATTAGCCATGGTCC |
| CyaREcoFor | GTTTTTTTTTTAATACGACTCACTATAGCTGAAAAACATAACCCATAAAATGC |
| CyaREcoRev | AAAAAATAAGCCCGTGTAAGGGAGATTACACAGG |
| deeplac | CGGGCCTCTTCGCTA |
| GcvBFor | GTTTTTTTTTTAATACGACTCACTATA G ACTTCCTGAGCCGGAACGAAAAG |
| GcvBRev | AAAAAAAAGCACCGCAATTAGGCGGTG |
| zeopBADlacZ | TCAACTTGGCCATGGTTTAGTTCCTCACCTTGTCGTATTAGCGCTTCAGCCATACTTTTC |
| pnpFLAG Rev | GCCCCCCGCCGCAGCGGAGGGCAAATGGCAACCTTATTTATCGTCGTCATCTTTGTAGTCGATATCATGATCTTTATAATCACCGTCATGGTCTTTGTAGTCCTCGCCCTGTTCAGCAGC |
| pnp606FLAG Rev | GCCCCCCGCCGCAGCGGAGGGCAAATGGCAACCTTATTTATCGTCGTCATCTTTGTAGTCGATATCATGATCTTTATAATCACCGTCATGGTCTTTGTAGTCCGCTTTCTCGCCGTCGGT |
| pnpccdB For | ACCAGTGCCGTAAGGTACTGTCTAAGAAAGAGAAAGGATATTACATTATATTCCCCAGAACATCAGG |
| pnpccdB Rev | GCCCGGTTAAAAGCCCCCCGCCGCAGCGGAGGGCAAATGGCAACCATAGGAACTTCAAGATCC |
| pnpinsccdB For | CCGTACGTGTTGTGTCTGAAATCACTGAATCCAACGGTTTATATTCCCCAGAACATCAGG |
| pnpinsccdB Rev | CGTCCATCAGCGCCAGAGACGCGCCGCACACGGAAGCCATATAGGAACTTCAAGATCC |
| pnpoligo | CACCGTACGTGTTGTGTCTGAAATCACTGAATCCAACGGTTCCTCTTCTATGGCTTCCGTGTGCGGCGCGTCTCTGGCGCTGATGGACG |
| pnpS437-9A oligo | CACCGTACGTGTTGTGTCTGAAATCACTGAATCCAACGGTGCCGCTGCTATGGCTTCCGTGTGCGGCGCGTCTCTGGCGCTGATGGACG |
| pnpS437-9A For | GAAATCACTGAATCCAACGGTGCCGCTGCTATGGCTTCCGTGTGCGGCGC |
| pnpS437-9A Rev | GCGCCGCACACGGAAGCCATAGCAGCGGCACCGTTGGATTCAGTGATTTC |
| pnpchrom For | CAGTGCCGTAAGGTACTG |
| pnpchrom Rev | GCCCCCCGCCGCAGCGGAGGGCAAATGGCAACCTTACTCG |
| pnpK606stop | GCCCGGTTAAAAGCCCCCCGCCGCAGCGGAGGGCAAATGGCAACCTTACGCTTTCTCGCCGTCGGT |
| pnpE550stop | GCCCGGTTAAAAGCCCCCCGCCGCAGCGGAGGGCAAATGGCAACCTTAAGAGATATCGCCACGCGG |
| pnp-ccdB For | ACCAGTGCCGTAAGGTACTGTCTAAGAAAGAGAAAGGATATTACATTATATTCCCCAGAACATCAGG |
| pnp-ccdB Rev | GCCCGGTTAAAAGCCCCCCGCCGCAGCGGAGGGCAAATGGCAACCATAGGAACTTCAAGATCC |
| RyhBFor | TATAGAATTCTAATACGACTCACTATAGCGATCAGGAAGACCCTC |
| RyhBRev | AAAAGCCAGCACCCGGCTGGCTAAG |
| RyhBRev9U | AAAAAAAAAGCCAGCACCCGGCTGGCTAAG |
| sodB-lacZ For | ACCTGACGCTTTTTATCGCAACTCTCTACTGTTTCTCCATATACGCACAATAAGGCTATTG |
| sodB-lacZ Rev | TAACGCCAGGGTTTTCCCAGTCACGACGTTGTAAAACGACTGGTAGTGCAGGTAATTCG |
|  |  |
| **Probes** |  |
| CyaR | TGGTTCCTGGTACAGCTAGCATTTTATGGGTTATG |
| GcvB | CCAGAACACGCATTCCGATAAAACTTTTCGTTCCGGCTCAGG |
| LeuX | CGTATTTCTACGGTTGATTTTG |
| MicA | CCAAAATTTCATCTCTGAATTCAGGGATGATGATAACAAATG |
| MgrR | CAGTAAACCGGCGGTGAATGCTTGCATGGATAGAT |
| RyhB | AAGTAATACTGGAAGCAATGTGAGCAATGTCGTGCTTTCAGGTTCTC |
| SsrA | CGCCACTAACAAACTAGCCTGATTAAGTTTTAACGCTTCA |
| 5s | ATGGGGTCAGGTGGGACCACCGCGCTACTGC |

**Table S4 Proteomics analyses of immunoprecipitated Hfq and PNPase**

Part 1: FLAG-antibody immunoprecipitated extracts from *Escherichia coli* expressing chromomosal PNPase-FLAG and the parental control.

| **Protein** | **Wild type,**  **exponential phase** | | **PNPase-FLAG,**  **exponential phase** | | **Wild type,**  **stationary phase** | | **PNPase-FLAG**  **stationary phase** | |
| --- | --- | --- | --- | --- | --- | --- | --- | --- |
|  | MASCOT | EMPAI* | MASCOT | EMPAI* | MASCOT | EMPAI* | MASCOT | EMPAI* |
| Hfq | < 42 | < 0.30 | 288 | 7.54 | 145 | 1.24 | 321 | 7.54 |
|  |  |  |  |  |  |  |  |  |
| Enolase | < 45 | < 0.08 | 1356 | 10.46 | 475 | 0.87 | 1548 | 15.24 |
|  |  |  |  |  |  |  |  |  |
| RhlB | < 45 | < 0.08 | 837 | 2.60 | < 41 | < 0.07 | 658 | 1.25 |
|  |  |  |  |  |  |  |  |  |
| Glycerol kinase | 870 | 1.47 | 1253 | 3.52 | 1604 | 9.45 | 1615 | 10.68 |
|  |  |  |  |  |  |  |  |  |
| PNPase | 121 | 0.09 | 1668 | 5.25 | 217 | 0.21 | 1485 | 5.25 |
|  |  |  |  |  |  |  |  |  |
| RNase E | < 42 | < 0.06 | 3537 | 19.06 | 102 | 0.06 | 2031 | 2.80 |
|  |  |  |  |  |  |  |  |  |
| RpoB | 675 | 0.35 | 2060 | 1.62 | 1127 | 0.57 | 2098 | 1.85 |
|  |  |  |  |  |  |  |  |  |
| RpoC | 471 | 0.26 | 2216 | 1.76 | 1190 | 0.86 | 1678 | 1.07 |

Immunoprecipitates were resolved on a SDS-PAGE gel, and the bands excised and analysed by LC/MS-MS by the proteomics facility, University of Cambridge, Department of Biochemistry.

*****EMPAI defined at 10^PAI^, where PAI - Nobsd/Nobsl, where Nobsd and Nobsl are then number of observed peptides per protein and the number of observable peptides per protein, respectively (Ishihama et al. 2005).

**Table S4 Proteomics analyses of immunoprecipitated Hfq and PNPase (continued)**

Part 2: Analysis of proteins immunoprecipitated with Hfq-FLAG in *Salmonella hfq-flag::km 3616* and parental control.

| **Protein** | **Wild type,**  **exponential phase** | | **Hfq-FLAG,**  **exponential phase** | | **Wild type,**  **stationary phase** | | **Hfq-FLAG**  **stationary phase** | |
| --- | --- | --- | --- | --- | --- | --- | --- | --- |
|  | MASCOT | EMPAI* | MASCOT | EMPAI* | MASCOT | EMPAI* | MASCOT | EMPAI* |
| PNPase | 40 | 0.04 | 368 | 0.46 | 66 | 0.09 | 606 | 1.03 |
|  |  |  |  |  |  |  |  |  |
| RNase E | Not detected | Not detected | 349 | 0.24 | Not detected | Not detected | 747 | 0.67 |
|  |  |  |  |  |  |  |  |  |
| Hfq | 89 | 1.95 | 340 | 24.71 | Not detected | Not detected | 440 | 129.39 |
|  |  |  |  |  |  |  |  |  |
| Pyruvate dehydrogenase E1 (known to interact with FLAG antibody) | 701 | 0.68 | 1334 | 1.81 | 920 | 1.03 | 1611 | 3.71 |

Part 3: Analysis of proteins complexes with Hfq. FLAG-antibody immunoprecipitated extracts from *Salmonella* expressing chromosomal Hfq-FLAG were resolved on a native gel, and PNPase bands identified by western blot with anti-PNPase antibodies. These bands were excised and analyzed by LC/MS-MS. The highest EMPAI scores are tabulated. The order of migration, from slow to fast, is Band 1, 2, 3, and 4.

| **Protein** | **Band 1** | **Band 2** | **Band 3** | **Band 4** |
| --- | --- | --- | --- | --- |
| Hfq | 10.42 | 32.70 | 10.42 | 43.18 |
| PNPase | 0.09 | 1.61 | 4.30 | 0.18 |
| S1 | - | - | 2.69 | - |
| Enolase | 0.26 | - | - | 0.88 |
| DNA polymerase β (RpoB) | 4.91 | 0.86 | - | - |
| DNA polymerase α (RpoA) | 3.73 | - | - | - |
| RNase E | 0.58 | - | - | - |
| Purine nucleoside phosphorylase | - | - | - | 3.75 |

**Supplementary Materials and Methods**

**Bacterial strains and plasmids**

All strains used in this study are derivatives of *E. coli* K12 strain MG1655. Strains and plasmids utilized are described in Supplemental Table S1. Oligonucleotides are listed in Supplemental Table S2 and were purchased from Integrated DNA Technologies, Inc. or Sigma-Aldrich Co., LLC. P1*vir* transductions were performed as described by Miller (Miller 1992). Plasmid pNRD466 was generated by site directed mutagenesis using the QuickChange II kit (Agilent Technologies) using primers pnpS437-9A For and Rev primers.

The *sodB'-'lacZ* fusion strain, NRD537, was constructed as described previously (Mandin and Gottesman 2009) via lambda Red recombinase-mediated recombineering using a PCR product generated by amplifying the *sodB* 5'UTR and first 9 codons from MG1655 genomic DNA using sodB-lacZ For and Rev primers, selecting for sucrose resistance, and screening for the correct insert by PCR and sequencing. The *dppA'-'lacZ* fusion was linked to zeocin resistance cassette by replacing the *kan ccdB* cassette in NM580 with a PCR product amplified from KM86 using zeopBADlacZ and deeplac primers by recombineering generating strain NRD1229. The zeocin resistance cassette in NRD1229 was subsequently replaced with a chloramphenicol resistance cassette by recombineering using a PCR product amplified from pKD3 using cat-P1 and cat-P2 primers.

NRD1021, NRD1022, and NRD1144 were constructed as follows. The *pnp* gene was first replaced with a PCR product containing a cassette encoding the kanamycin resistance gene and *ccdB* toxin gene by lamba Red recombinase-mediated allele replacement. The PCR product was amplified from CR201 genomic DNA using pnpccdB For and Rev primers. The *kan ccdB* cassette in the resulting strain, NRD1009, was subsequently replaced with PCR products generated by amplifying *pnp* from pNRD412 using pnpchrom For and pnpE550stop primers (NRD1021), pNRD466 using pnpchrom For and Rev primers (NRD1022), or pNRD412 using pnpchrom For and pnpK606stop primers (NRD1144). Successful recombinants were selected on arabinose plates, which induce CcdB toxin expression, and screened by PCR and sequencing.

Strain NRD1243, which has the chromosomal copy of *pnp* exchanged with a FLAG-tagged encoding allele, was generated by replacing the *kan ccdB* cassette of NRD1009 via lambda Red recombinase-mediated allele replacement with a PCR product amplified from plasmid pNRD412 using pnpchrom For and pnpFLAG Rev primers. Successful recombinants were isolated as described above.

Strain NRD1246 encoding PNPaseΔS1 FLAG was generated in a multi-step process. The *kan ccdB* cassette in NRD1009 was replaced by lambda Red recombinase-mediated allele replacement with a PCR product generated from plasmid pNRD466 using primers pnpchrom For and pnp606FLAG rev to generate strain NRD1249, which has codons 437, 438, and 439 of *pnp* mutated. Codons 437-439 were subsequently replaced with a *kan ccdB* cassette using a PCR product generated from CR201 using pnpinsccdB For and Rev primers yielding strain NRD1268. The wild type sequence of codons 437 to 439 was then inserted by lambda Red mediated recombination using pnpoligo DNA to generate strain NRD1246.

Strain NRD1247 was constructed as follows. Codons 437 to 439 of *pnp::3XFLAG* from strain NRD1243 were replaced with a *kan ccdB* cassette as described above, and the mutations that result in the S437A S438A and S439A substitutions were introduced by replacing the *kan ccdB* cassette via lambda red recombinase mediated gene replacement using the pnpS437-9Aoligo DNA.

**β-galactosidase assays**

β-galactosidase activity assays were performed as described in Materials and Methods.

**Supplementary References**

Baba T, Ara T, Hasegawa M, Takai Y, Okumura Y, Baba M, Datsenko KA, Tomita M, Wanner BL, Mori H. 2006. Construction of Escherichia coli K-12 in-frame, single-gene knockout mutants: the Keio collection. *Molecular systems biology* **2**: 2006 0008.

Battesti A, Majdalani N, Gottesman S. 2015. Stress sigma factor RpoS degradation and translation are sensitive to the state of central metabolism. *Proceedings of the National Academy of Sciences of the United States of America* **112**: 5159-5164.

Datsenko KA, Wanner BL. 2000. One-step inactivation of chromosomal genes in Escherichia coli K-12 using PCR products. *Proceedings of the National Academy of Sciences of the United States of America* **97**: 6640-6645.

De Lay N, Gottesman S. 2009. The Crp-activated small noncoding regulatory RNA CyaR (RyeE) links nutritional status to group behavior. *Journal of bacteriology* **191**: 461-476.

De Lay N, Gottesman S. 2011. Role of polynucleotide phosphorylase in sRNA function in Escherichia coli. *Rna* **17**: 1172-1189.

Guillier M, Gottesman S. 2006. Remodelling of the Escherichia coli outer membrane by two small regulatory RNAs. *Molecular microbiology* **59**: 231-247.

Ishihama Y, Oda Y, Tabata T, Sato T, Nagasu T, Rappsilber J, Mann M. 2005. Exponentially modified protein abundance index (emPAI) for estimation of absolute protein amount in proteomics by the number of sequenced peptides per protein. *Molecular & cellular proteomics : MCP* **4**: 1265-1272.

Mandin P, Gottesman S. 2009. A genetic approach for finding small RNAs regulators of genes of interest identifies RybC as regulating the DpiA/DpiB two-component system. *Molecular microbiology* **72**: 551-565.

Mandin P, Gottesman S. 2010. Integrating anaerobic/aerobic sensing and the general stress response through the ArcZ small RNA. *The EMBO journal* **29**: 3094-3107.

Masse E, Escorcia FE, Gottesman S. 2003. Coupled degradation of a small regulatory RNA and its mRNA targets in Escherichia coli. *Genes & development* **17**: 2374-2383.

Miller JH. 1992. *A Short Course in Bacterial Genetics*. Cold Spring Harbor Laboratory Press, Cold Spring Harbor, NY.

Mohanty BK, Kushner SR. 2010. Processing of the Escherichia coli leuX tRNA transcript, encoding tRNA(Leu5), requires either the 3'-->5' exoribonuclease polynucleotide phosphorylase or RNase P to remove the Rho-independent transcription terminator. *Nucleic acids research* **38**: 597-607.

Moon K, Gottesman S. 2011. Competition among Hfq-binding small RNAs in Escherichia coli. *Molecular microbiology* **82**: 1545-1562.
